# Quantitative CT Geometry of the Patent Ductus Arteriosus in Infants with Congenital Heart Disease: Association with Aortic Arch Obstruction or Hypoplasia

**DOI:** 10.3390/jcm15208017

**Published:** 2026-10-16

**Authors:** Marian Pop, Liel Bargutin, Răzvan-Andrei Licu, Marian Rotariu, Diana-Ramona Iurian, Tania Negovan, Horațiu Suciu

**Affiliations:** 1 Faculty of Medicine in English, George Emil Palade University of Medicine, Pharmacy, Science and Technology of Târgu Mureș, 540142 Târgu Mureș, Romania; 2 Radiology and Medical Imaging Laboratory, Mureș County Emergency Clinical Hospital, 540136 Târgu Mureș, Romania; 3 Emergency Institute for Cardiovascular Diseases and Transplantation of Târgu Mureș, 540139 Târgu Mureș, Romania; 4 Doctoral School of Medicine and Pharmacy, George Emil Palade University of Medicine, Pharmacy, Science and Technology of Târgu Mureș, 540142 Târgu Mureș, Romania; 5 Department of Surgery IV, George Emil Palade University of Medicine, Pharmacy, Science and Technology of Târgu Mureș, 540142 Târgu Mureș, Romania; 6 Clinic of Cardiovascular Surgery, Emergency Institute for Cardiovascular Diseases and Transplantation of Târgu Mureș, 540139 Târgu Mureș, Romania

**Keywords:** patent ductus arteriosus, congenital heart disease, aortic coarctation, aortic arch hypoplasia, computed tomography angiography, morphometry, ductal length-to-diameter index

## Abstract

**Background/Objectives:** Quantitative patent ductus arteriosus (PDA) geometry in congenital heart disease (CHD), particularly in relation to aortic arch abnormalities, is incompletely characterized. We evaluated CT-derived PDA geometry. **Methods:** This retrospective exploratory single-center study included 168 infants with CHD and a PDA on cardiovascular CT. Aortic-end, pulmonary-end, and mid-duct diameters and ductal length were measured. The Ductal Length-to-Diameter Index (LDI), selected during exploratory analysis, was defined as ductal length divided by the mean end diameter. Seventy-three patients had CT-documented aortic arch obstruction or hypoplasia. **Results:** LDI was lower in patients with arch obstruction/hypoplasia than in those without these findings (2.04 [1.39–2.84] vs. 3.51 [2.23–4.89]; *p* < 0.001; rank-biserial *r* = −0.473) and remained associated after adjustment for age, weight, and sex (OR per +1 LDI, 0.534; 95% CI, 0.412–0.693; *p* < 0.001). The association remained robust with a conservative definition restricted to coarctation or interrupted aortic arch. LDI had an interobserver ICC of 0.843 (95% CI, 0.685–0.954), with wide limits of agreement (−1.98 to +2.45). **Conclusions:** CT-documented aortic arch obstruction or hypoplasia is associated with lower LDI. This exploratory geometric association warrants prospective external validation before clinical use.

## 1. Introduction

The ductus arteriosus connects the pulmonary artery to the descending aorta in fetal life and normally closes after birth. Persistent patency may be an isolated lesion or an integral component of complex congenital heart disease (CHD), particularly when systemic or pulmonary blood flow is duct-dependent [1,2]. Beyond hemodynamic significance, ductal caliber, length, origin, insertion, orientation, and tortuosity can influence surgical and transcatheter planning, device or stent selection, and assessment of the duct’s relationship with adjacent vessels [3,4,5].

Patent ductus arteriosus (PDA) morphology is commonly described using the angiographic classification of Krichenko et al. [6], developed primarily for isolated PDA. Neonatal and complex congenital ductal anatomy is more diverse: fetal-type morphology, marked tortuosity, unusual origins and insertions, and postnatal morphological changes have been described in premature infants and duct-dependent CHD [3,7,8,9]. These observations suggest that categorical description can be complemented by continuous geometric measurements that preserve length and caliber information.

Cardiovascular computed tomography (CT) provides high-spatial-resolution three-dimensional assessment of extracardiac anatomy in CHD and is particularly useful when the great arteries, aortic arch, pulmonary arteries, or other vascular structures require detailed delineation [10,11,12,13]. CT-derived PDA measurements correlate with catheter angiography, support stent planning, and can depict cross-sectional features not evident on projection imaging [4,5,14]. CT is also well suited to identifying associated aortic arch abnormalities [15].

The relationship between the ductus and aortic arch obstruction is of particular developmental and clinical interest. Coarctation is typically juxtaductal, and histological studies demonstrate extension of ductal tissue into the adjacent aortic wall [16,17,18]. A widely patent ductus can also alter the anatomical and hemodynamic expression of neonatal arch obstruction, prompting prior work on aortic and echocardiographic predictors of coarctation while the ductus remains open [19,20,21,22]. Whether the geometry of the PDA itself differs systematically with arch obstruction has received substantially less quantitative study.

Most prior quantitative PDA imaging work has focused on procedural planning, especially ductal stenting or device closure, rather than on the relationship between ductal geometry and associated congenital cardiovascular anatomy [4,5,14]. The Ductal Length-to-Diameter Index (LDI) expresses ductal length relative to the mean of its two end diameters, not to aortic diameter. A lower value may reflect shorter length, larger end caliber, or both; the developmental ductus–isthmus relationship provides anatomical plausibility but does not establish a causal mechanism. LDI may standardize description of ductal geometry, but its incremental value for treatment selection or prognosis remains unproven.

We evaluated quantitative CT geometry of the PDA in infants with CHD according to CT-documented aortic arch obstruction or hypoplasia. LDI was selected during exploratory analysis and was not prospectively predefined; the findings are hypothesis-generating rather than prospectively confirmatory. Secondary objectives were to assess other geometric characteristics and broader physiological groups.

## 2. Materials and Methods

### 2.1. Study Design and Population

This retrospective, single-center study assessed 174 records of patients with CHD who underwent clinically indicated cardiovascular CT angiography at the Emergency Institute for Cardiovascular Diseases and Transplantation of Târgu Mureș, Romania, between 9 January 2018 and 30 July 2026. Patients were eligible when a PDA was identified and measurable on CT. Five postoperative examinations in which previous surgery altered native anatomy relevant to ductal morphometry and one duplicate record were excluded, leaving 168 unique patients and one examination per patient. No additional eligibility criteria were applied. Demographic and clinical variables, referral and CT diagnoses, and radiation-dose data were retrieved from the available records.

### 2.2. CT Acquisition and Image Reconstruction

Cardiovascular CT angiography was performed on a SOMATOM Definition AS 64-slice (Siemens Healthineers, Forchheim, Germany), Revolution HD 64-slice, or Revolution Frontier 128-slice (GE HealthCare, Chicago, IL, USA) scanner using prospective electrocardiogram (ECG) gating. Tube voltage was generally 80 kV and adapted to body habitus, with acquisition parameters clinically optimized for patient size; gantry rotation time was 0.3 s. Iodinated contrast material (320, 350, or 400 mg I/mL) was administered according to clinical protocol, with bolus tracking for timing. Images were reconstructed at 0.6 or 0.625 mm using SAFIRE (Siemens Healthineers) or ASiR (GE HealthCare). Measurements were performed in RadiAnt DICOM Viewer, version 2020 (Medixant, Poznań, Poland).

### 2.3. PDA Measurements

**Figure 1:**
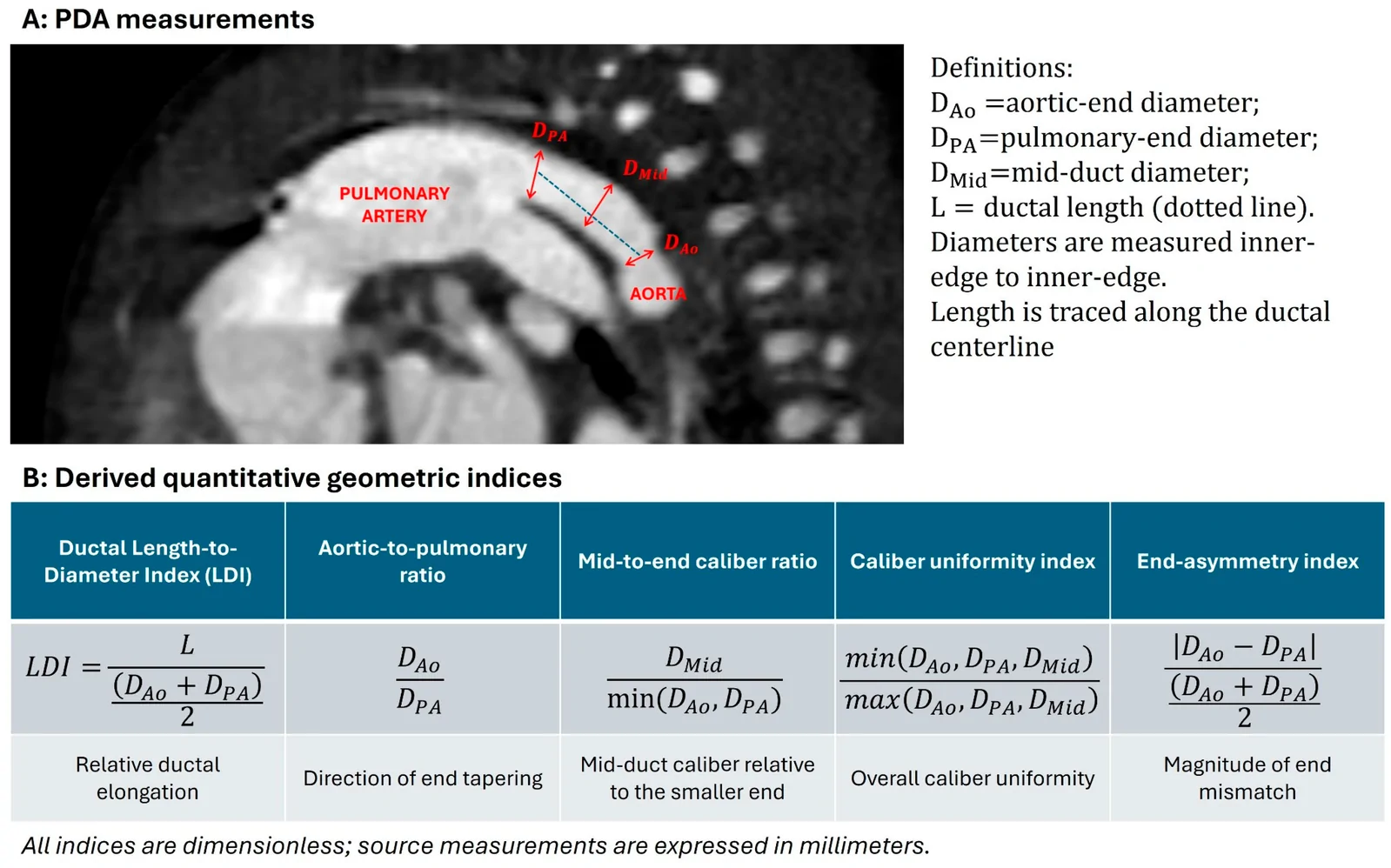
CT-derived PDA measurements and dimensionless geometric indices. (**A**) Representative contrast-enhanced multiplanar CT reformation showing the aortic-end diameter (D_Ao), pulmonary-end diameter (D_PA), mid-duct diameter (D_Mid), and ductal length (L). Diameters were measured from inner edge to inner edge; L was traced along the ductal centerline (dotted line). (**B**) Formulas for the Ductal Length-to-Diameter Index (LDI), aortic-to-pulmonary ratio, mid-to-end caliber ratio, caliber uniformity index, and end-asymmetry index. Source measurements are in millimeters.

PDA measurements were obtained on contrast-enhanced multiplanar reformations aligned with the ductal axis. Aortic-end diameter (D_Ao), pulmonary-end diameter (D_PA), and mid-duct diameter (D_Mid) were measured inner edge to inner edge on planes adjusted to the local ductal orientation to minimize obliquity. Ductal length (L) was traced from the aortic to pulmonary attachment along the ductal centerline; in tortuous ducts, consecutive segments were summed. Measurements were expressed in millimeters. Missing measurements were not imputed, and patients were excluded only from analyses requiring the unavailable variable (Figure 1).

### 2.4. Derived Quantitative Geometry Indices

Continuous geometric indices were calculated from the source measurements. The principal parameter, the Ductal Length-to-Diameter Index, was LDI = L/[(D_Ao + D_PA)/2]. D_Ao and D_PA denote the ductal diameters at the aortic and pulmonary attachments, respectively. The arithmetic mean of the two ductal end diameters was used as a symmetric, equally weighted caliber reference; this pragmatic normalization has not been established as optimal. Secondary indices were D_Ao/D_PA, D_Mid/min(D_Ao, D_PA), min(D_Ao, D_PA, D_Mid)/max(D_Ao, D_PA, D_Mid), and |D_Ao − D_PA|/[(D_Ao + D_PA)/2], representing end ratio, mid-to-end caliber, caliber uniformity, and end asymmetry, respectively. No threshold-based categorization of these indices was used in the principal analysis.

### 2.5. Diagnostic Classification

For the principal analysis, patients were classified by CT-documented aortic arch obstruction or hypoplasia using the CT diagnosis as the primary anatomical source. The case group included coarctation, interrupted aortic arch, and explicitly documented arch or isthmic hypoplasia. Arch/isthmic hypoplasia required explicit documentation in the original CT report; no uniform study-wide z-score or aortic-ratio threshold was applied. Descriptions limited to low-normal or borderline caliber, kinking, or decalibration without explicit hypoplasia, coarctation, or obstruction remained in the comparison group. Other arch variants were not considered obstructive unless an obstructive or hypoplastic lesion was also documented. Referral diagnosis was retained as clinical information but was not used for primary adjudication.

For secondary analyses, diagnoses were assigned to four mutually exclusive categories: arch obstruction/hypoplasia, other systemic outflow obstruction, pulmonary duct-dependent disease, and other CHD. The first two were combined for the physiology-based analysis. Assignments were based on CT diagnosis without reference to PDA indices, and ambiguous wording was manually adjudicated using the same explicit-terminology rule. De-identified patient-level assignments are provided in Table S1.

### 2.6. Interobserver Reproducibility Assessment

Interobserver reproducibility was evaluated in 30 randomly selected patients drawn from the first 150 consecutively assessed examinations, before the cohort was extended to 174 records. Measurements were repeated independently by a second radiologist with >3 years of cardiovascular imaging experience; the primary reader had >10 years of experience. The second observer was blinded to all clinical information, the study’s aortic arch classification, and the first observer’s measurements; anatomical findings remained visible on CT. Each reader measured D_Ao, D_PA, D_Mid, and L using identical definitions, and the derived indices were calculated independently. A fixed rule-based categorical profile was also derived from each reader’s measurements and atypia assessment; the reader-assigned and algorithmic profiles are provided in Table S2 and the categorical sensitivity analyses in Table S3.

The rule-based profile classified ducts as window-short, hourglass, tubular, conical-aortic, conical-pulmonary, mixed, or complex/atypical. Complex or atypical morphology overrode the remaining categories when a reverse angle, serpentine course, filiform caliber, or anomalous origin/insertion was documented; otherwise, the profile was determined sequentially from fixed length and diameter-ratio thresholds. Exact rules are reported in Table S5, and the case-level profiles of both readers in Table S2, so that categorical agreement can be reproduced without adding a second classification framework to the main analysis.

### 2.7. Statistical Analysis

Continuous variables are reported as median [interquartile range (IQR)] and categorical variables as *n* (%). Continuous groups were compared with the Mann–Whitney U test and categorical groups with Fisher’s exact test. LDI was selected during exploratory analysis and was not prospectively predefined; the findings are hypothesis-generating rather than prospectively confirmatory. The index was fixed before final database lock, and between-group effect size was summarized by rank-biserial correlation.

The principal association was evaluated using multivariable logistic regression with CT-documented arch obstruction/hypoplasia as the outcome and LDI as the predictor, adjusted for log(age + 1), body weight, and sex. Age and weight accounted for postnatal timing and body size, respectively; sex was included as a demographic covariate. These covariates were retained irrespective of statistical significance, and no automated or stepwise selection was used. Results are reported as odds ratios (ORs) with 95% confidence intervals (CIs). Seven secondary morphometric parameters—absolute length, both end diameters, aortic-to-pulmonary ratio, mid-to-end caliber ratio, caliber uniformity, and end asymmetry—were evaluated with Holm correction across this family of tests. Analyses used complete cases without imputation.

LDI was compared across systemic/arch obstruction, pulmonary duct-dependent disease, and other CHD using Kruskal–Wallis testing with Holm-adjusted pairwise Mann–Whitney tests. Robustness analyses used restricted and broader comparator groups, a conservative case definition limited to coarctation/interrupted arch with explicit hypoplasia cases either included in the comparator or excluded, direct comparison of explicit hypoplasia with classic obstruction, and alternative adjudication of borderline CT wording. A component model entered log(PDA length) and log(mean endpoint diameter) separately. Exploratory analyses assessed LDI nonlinearity with a cubic B-spline, compared otherwise identical adjusted LDI and length models by the Akaike information criterion (AIC), estimated areas under the receiver operating characteristic curve (AUCs) without formal between-AUC testing, and examined age-stratified results and interaction. Full specifications and outputs are provided in Table S4 and Supplementary Code S1.

The component model contained log(PDA length), log(mean endpoint diameter), log(age + 1), weight, and sex. The spline analysis used a cubic B-spline with 4 degrees of freedom (degree 3) and was compared with the linear LDI model by likelihood-ratio test. The post hoc age analysis used strata <10 and ≥10 days and tested an LDI × age-stratum interaction adjusted for weight and sex; age was not treated as a surrogate for prostaglandin E1 (PGE1) exposure.

Interobserver reproducibility was assessed using a two-way random-effects, absolute-agreement, single-measure intraclass correlation coefficient [ICC(A,1)], Lin’s concordance correlation coefficient (CCC), Bland–Altman bias and 95% limits of agreement, and mean absolute error (MAE). Categorical agreement was summarized by unweighted Cohen’s κ and exact agreement. Confidence intervals for agreement estimates were obtained from 10,000 paired patient-level percentile bootstrap resamples using fixed seed 20260818. Tests were two-sided with *p* < 0.05; Holm-adjusted *p* values were used where specified. Analyses were performed in Python 3.13.5 (Python Software Foundation, Wilmington, DE, USA) using the package versions listed in Supplementary Code S1.

### 2.8. Use of Generative Artificial Intelligence

Generative AI was used as an assistive tool for statistical planning, code generation and checking, literature organization, and manuscript drafting/editing: ChatGPT (GPT-5.6 Sol, OpenAI, San Francisco, CA, USA) during development and Claude Opus 5 High (Anthropic, San Francisco, CA, USA) for a secondary audit of statistical reproducibility, reference accuracy, and manuscript consistency. All numerical results and references were checked by the authors against source data or primary sources. AI did not generate, alter, impute, or classify original patient data; all analytical decisions and final content remained under author control.

## 3. Results

### 3.1. Study Population

**Figure 2:**
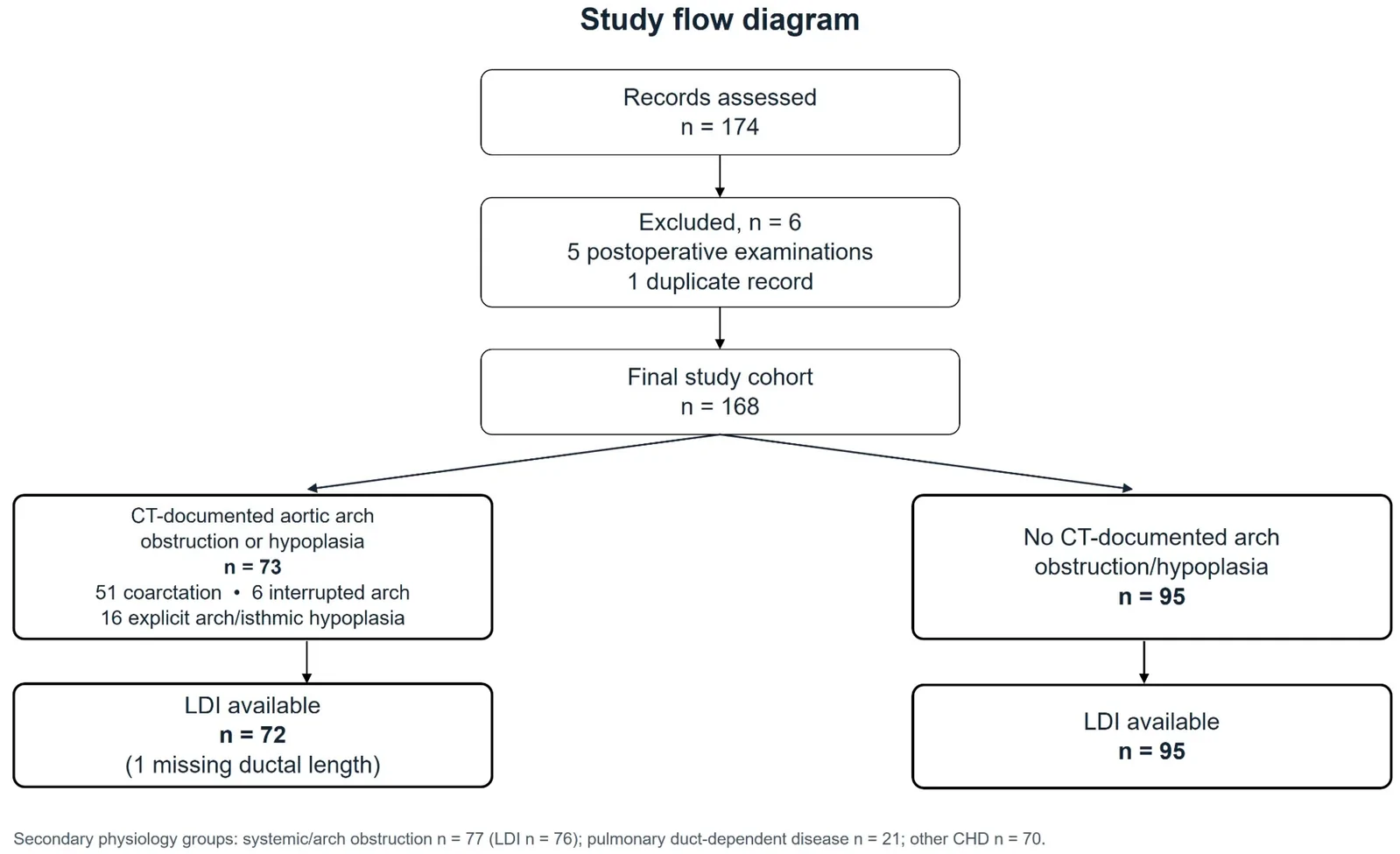
Study flow and diagnostic classification. Of 174 records assessed, six were excluded (five postoperative examinations and one duplicate record), leaving 168 unique patients in the final cohort. Seventy-three met the report-based CT-diagnosis criteria for CT-documented aortic arch obstruction or hypoplasia, and 95 did not. PDA length was unavailable in one patient from the arch obstruction/hypoplasia group, resulting in 167 patients with calculable LDI. For the secondary physiology-based analysis, patients were categorized as systemic/arch obstruction (*n* = 77; LDI available in 76), pulmonary duct-dependent disease (*n* = 21), or other congenital heart disease (*n* = 70).

Of 174 records assessed, five postoperative examinations and one duplicate were excluded, leaving 168 patients (Figure 2). Median age was 7 [4,5,6,7,8,9,10,11,12,13] days; 156/168 (92.9%) were neonates, and 94 (56.0%) were male. Seventy-three patients had CT-documented arch obstruction/hypoplasia (51 coarctation, six interrupted arch, 16 explicit arch/isthmic hypoplasia) and 95 did not. LDI was available in 167 patients because ductal length was missing in one case. Baseline characteristics were otherwise similar between groups (Table 1).

### 3.2. Main Quantitative PDA Geometry Analysis

**Figure 3:**
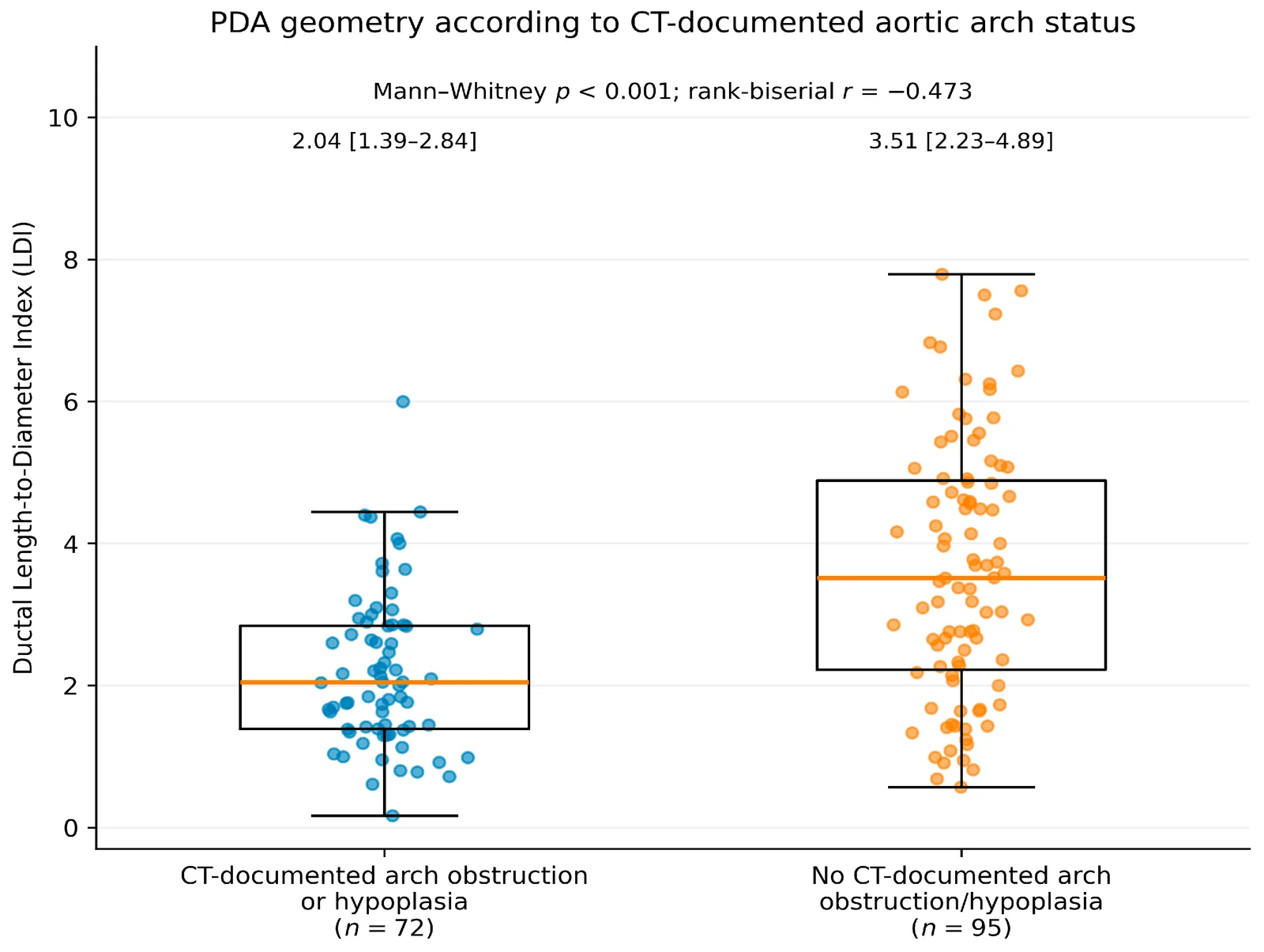
Ductal Length-to-Diameter Index according to CT-documented aortic arch status. Individual observations are superimposed on boxplots (blue circles, arch obstruction/hypoplasia group; orange circles, comparison group); boxes represent the interquartile range, the orange horizontal line indicates the median, and whiskers extend to the most extreme values within 1.5 × IQR of the box. LDI was significantly lower in patients with CT-documented aortic arch obstruction or hypoplasia (2.04 [1.39–2.84]) than in the comparison group (3.51 [2.23–4.89]; Mann–Whitney *p* < 0.001; rank-biserial *r* = −0.473). LDI was available in 167 patients (72 in the arch obstruction/hypoplasia group and 95 in the comparison group).

LDI was lower in patients with CT-documented arch obstruction/hypoplasia than in those without these findings (2.04 [1.39–2.84] vs. 3.51 [2.23–4.89]; *p* = 1.77 × 10^−7^; rank-biserial *r* = −0.473; Figure 3). In 164 complete cases, the association persisted after adjustment for age, weight, and sex (OR per +1 LDI, 0.534; 95% CI, 0.412–0.693; *p* = 2.32 × 10^−6^).

### 3.3. Secondary Quantitative PDA Characteristics

The arch obstruction/hypoplasia group also had a shorter absolute PDA, a larger pulmonary-end diameter, and a lower aortic-to-pulmonary diameter ratio after Holm correction; the other secondary indices did not differ significantly (Table 2).

### 3.4. Geometry According to Congenital Cardiovascular Physiology

LDI differed across systemic/arch obstruction (*n* = 76; 2.04 [1.38–2.84]), pulmonary duct-dependent disease (*n* = 21; 4.62 [2.76–5.43]), and other CHD (*n* = 70; 3.49 [2.20–4.69]; Kruskal–Wallis *p* = 1.22 × 10^−7^). After Holm correction, systemic/arch obstruction differed from pulmonary duct-dependent disease (adjusted *p* = 0.00027) and other CHD (adjusted *p* = 1.03 × 10^−6^), whereas pulmonary duct-dependent disease and other CHD did not differ significantly (adjusted *p* = 0.258; Figure S1).

### 3.5. Sensitivity and Robustness Analyses

The principal association remained robust across alternative definitions (Table S4). Restricting the comparator to other CHD yielded *p* = 6.62 × 10^−7^ (*r* = −0.484), and the broader systemic/arch definition yielded *p* = 2.35 × 10^−8^ (*r* = −0.503). LDI did not differ significantly between the 16 explicit hypoplasia cases and coarctation/interrupted arch (2.11 [1.45–2.74] vs. 1.94 [1.37–2.87]; *p* = 0.887), while comparison of coarctation/interrupted arch with all remaining patients, including the 16 hypoplasia cases in the comparator, preserved the association (56 vs. 111 patients with LDI; *p* = 2.19 × 10^−5^; *r* = −0.403). Alternative adjudication of borderline CT wording produced the same conclusion (Table S4). Excluding the 16 hypoplasia cases entirely also preserved the association against the original comparator (56 vs. 95 patients with LDI; *p* = 1.75 × 10^−6^; *r* = −0.467).

In the component model, both shorter ductal length (OR per doubling, 0.442; 95% CI, 0.276–0.708; *p* = 0.00067) and larger mean endpoint caliber (OR per doubling, 3.26; 95% CI, 1.67–6.36; *p* = 0.00053) were associated with arch obstruction/hypoplasia. A spline did not improve fit over a linear LDI term (*p* = 0.221). The adjusted LDI model had a lower AIC than the corresponding length model (202.52 vs. 222.68), and LDI remained associated in a joint model containing both terms (*p* = 4.09 × 10^−5^), whereas log(length) did not (*p* = 0.262). Exploratory AUCs in the same complete-case sample were 0.728 for LDI and 0.647 for length; no formal AUC comparison was performed.

In the post hoc age analysis, LDI remained lower in the arch obstruction/hypoplasia group both below 10 days (1.84 [1.39–2.84] vs. 3.18 [2.00–4.62]; *p* = 7.43 × 10^−5^) and at ≥10 days (2.32 [1.40–2.93] vs. 4.02 [2.69–5.72]; *p* = 0.00072), with no evidence of interaction by age stratum (*p* = 0.744). These analyses were exploratory and were not used as a surrogate assessment of PGE1 exposure.

### 3.6. Interobserver Reproducibility

In the 30-case interobserver substudy (Table S2), ICC(A,1) was 0.911, 0.865, and 0.905 for aortic-end, pulmonary-end, and mid-duct diameters, respectively; absolute length was less reproducible (ICC, 0.774). LDI had an ICC of 0.843 (95% CI, 0.685–0.954), but absolute agreement was limited (bias, +0.24; 95% limits of agreement, −1.98 to +2.45; MAE, 0.64; Figure S2). Rule-based categorical profiles showed moderate agreement (κ = 0.460; exact agreement 56.7%; Table 3), whereas reader-assigned profiles showed higher agreement (κ = 0.664; exact agreement 73.3%). Additional categorical sensitivity analyses are provided in Table S3.

## 4. Discussion

This study shows that PDA geometry differs in infants with CT-documented aortic arch obstruction or hypoplasia. LDI was substantially lower in the affected group and remained associated after adjustment for age, weight, and sex. The same group also had a shorter absolute ductus, a larger pulmonary-end diameter, and a lower aortic-to-pulmonary diameter ratio, whereas aortic-end diameter did not differ significantly. The pattern therefore reflects both relative ductal shortening and pulmonary-to-aortic caliber predominance. Because the study is observational, it should be interpreted as an anatomical association rather than evidence of a causal developmental mechanism.

The association is anatomically plausible because the ductus and aortic isthmus are closely related developmentally and histologically. Ductal tissue extends into the juxtaductal aortic wall in coarctation [16,17,18], and persistent ductal patency can mask or modify the neonatal expression of arch obstruction [19,20,21,22]. Previous studies have therefore focused on aortic and echocardiographic predictors of coarctation while the PDA remains open. Our analysis approaches the same interaction from the opposite direction: the ductus itself appears to carry quantitative geometric information associated with the arch phenotype. These data do not demonstrate that incorporation of ductal tissue into the aortic wall causes ductal shortening.

The physiology-based analysis points in the same direction. LDI was lowest in the systemic/arch obstruction group and highest in pulmonary duct-dependent disease, with other CHD intermediate. This pattern raises the possibility that ductal geometry reflects not only a local juxtaductal relationship but also broader fetal or neonatal flow conditions. However, the non-arch systemic outflow group was small, and the present data cannot distinguish developmental anatomy from flow-mediated remodeling.

Robustness analyses reduced concern that the result was driven by the expanded hypoplasia definition. LDI did not differ significantly between explicit hypoplasia and coarctation/interrupted arch, and the association persisted when hypoplasia cases were excluded entirely. More inclusive adjudication of borderline reports also produced the same conclusion. This matters because CT wording such as low-normal caliber or decalibration can be clinically ambiguous. Nevertheless, LDI should not be interpreted as a diagnostic threshold or a finding specific for coarctation; other disease processes can mimic arch obstruction [23], and external validation is required. No clinical LDI threshold is proposed, as diagnostic performance requires prospective evaluation against an independent reference standard.

The component analysis helps interpret LDI and its potential confounders. Both shorter ductal length and larger mean endpoint caliber contributed to the lower index. PGE1 may increase ductal caliber and thereby lower LDI even at a fixed length. Individual exposure, dose, and treatment duration were unavailable, so neither age adjustment nor component analyses exclude this influence. Because PGE1 is used in both systemic and pulmonary duct-dependent disease, the net between-group effect cannot be established without individual treatment data. The lower AIC and numerically higher exploratory AUC of LDI relative to absolute length support further study as a compact geometric descriptor, without establishing clinical utility.

Quantitative CT characterization should be viewed as complementary to established morphological descriptions. Krichenko morphology remains widely used [6], but fetal-type, tortuous, and complex ductal configurations in neonatal CHD illustrate the limitations of assigning a multidimensional structure to a single category [3,7,8,9]. CT is well suited to continuous measurement and three-dimensional assessment [4,5,10,11,12,13,14,15,24,25], while acquisition strategy remains important for motion-sensitive vascular measurements [26]. The indices used here preserve different aspects of geometry—relative length, directional tapering, mid-duct caliber, uniformity, and end asymmetry—without proposing a replacement classification.

This quantitative approach differs from creating another categorical PDA classification. LDI is intentionally simple and dimensionless, while the accompanying indices separate tapering, mid-duct caliber, uniformity, and endpoint asymmetry. Such measures can be calculated from routine CT multiplanar reformations and retain continuous information that is lost when a duct is forced into a single morphology category. They may also provide a common numerical language for future comparisons across imaging, catheterization, and three-dimensional planning studies.

Detailed ductal anatomy also has established procedural relevance. Ductal stenting and transcatheter closure depend on origin, length, caliber, curvature, insertion, and adjacent vascular anatomy [3,4,5,9,14,27,28,29,30,31], while surgery remains appropriate in selected anatomical and clinical settings [32]. CT-based measurements can therefore be clinically useful even when no single index is used diagnostically. More broadly, anatomy-directed management is also central to uncommon pediatric congenital structural lesions [33]. The present study did not evaluate procedural outcomes, so no therapeutic implication should yet be assigned to LDI; its procedural value requires dedicated correlation with stent, device, or surgical outcomes.

The interobserver results support measurement-based characterization. The three diameter measurements showed good-to-excellent agreement, and LDI retained a favorable ICC despite the lower reproducibility of manually traced length. Rule-based categorical agreement was only moderate, although reader-assigned profiles performed better. ICC and κ quantify different constructs, so these data do not establish formal statistical superiority of continuous over categorical morphology. The results support further standardization of measurement-based characterization, consistent with recent CT-derived planning work [34].

Despite an ICC of 0.843, LDI showed wide Bland–Altman limits of agreement (−1.98 to +2.45), limiting individual threshold interpretation. Tracing a tortuous duct introduces more reader choice than a local diameter measurement. These interobserver results do not establish longitudinal test–retest repeatability or a smallest detectable biological change.

Several limitations are important. The study was retrospective, single-center, and restricted to clinically referred infants with CHD, limiting generalizability to isolated PDA or unselected neonatal populations. LDI was identified during exploratory work and fixed before final database lock; the findings are therefore hypothesis-generating rather than prospectively confirmatory. Individual PGE1 exposure, gestational age, and prematurity were unavailable. PGE1 is a particularly important potential confounder for the caliber component, while chronological age cannot substitute for gestational age. The cohort was diagnostically heterogeneous and included relatively small physiological subgroups. Report-based hypoplasia classification lacked a uniform numerical criterion, limiting reproducibility across institutions.

The study also spanned more than eight years and three CT platforms, with a dose-length product (DLP) range of 3–295 mGy·cm, introducing acquisition, reconstruction, and dose heterogeneity. Ductal length was manually traced rather than derived from an automated centerline, and its lower reproducibility may propagate into LDI, particularly in tortuous ducts. Curvature, tortuosity, angular measures, and automated three-dimensional segmentation were not assessed [4,5,34]. The categorical profile was a study-specific descriptor rather than a validated replacement for established classifications. Finally, the interobserver sample was drawn from the first 150 examinations before cohort extension, so later examinations had no opportunity for selection, and procedural or surgical outcomes were unavailable.

Spatial resolution is another consideration because some neonatal ductal diameters approach the reconstructed slice thickness. Measurements were made on multiplanar reformations with local plane adjustment, but partial-volume effects cannot be eliminated. Scanner-specific reconstruction and the absence of automated segmentation may therefore contribute additional measurement variability. These technical limitations further support treating the present indices as research descriptors rather than diagnostic cut-offs.

Prospective multicenter validation should prespecify LDI, use blinded measurements and standardized arch adjudication, and record gestational age and PGE1 exposure. Assuming a conservative standardized difference of 0.50 in log(LDI), 64 evaluable patients per group would provide 80% power at a two-sided α of 0.05; approximately 144 recruits would allow 10% non-evaluable data with balanced allocation. This is an illustrative association-replication target; diagnostic or clinical-outcome validation requires separate planning.

## 5. Conclusions

In infants with CHD, CT-documented aortic arch obstruction or hypoplasia was associated with lower LDI, reflecting differences in ductal length and end caliber. For prospective replication using clinically indicated CT, a minimal measurement set comprises ductal centerline length and aortic-end, pulmonary-end, and mid-duct diameters. LDI remains a research descriptor; clinical use requires external validation with standardized arch assessment, documented PGE1 exposure, and independent clinical outcomes.

## Figures and Tables

**Table 1:** Baseline characteristics of the study population according to CT-documented aortic arch status.

Variable	Overall (*n* = 168)	CT-Documented Arch Obstruction or Hypoplasia (*n* = 73)	No CT-Documented Arch Obstruction/ Hypoplasia (*n* = 95)	*p* Value
Age, days	7.0 [4.0–13.0]	6.0 [4.0–11.0]	8.0 [5.0–14.0]	0.331
Neonatal age (≤28 days), *n* (%)	156 (92.9)	69 (94.5)	87 (91.6)	0.555
Male sex, *n* (%)	94 (56.0)	41 (56.2)	53 (55.8)	1.000
Weight, kg	3.07 [2.80–3.50]	3.00 [2.77–3.48]	3.15 [2.81–3.65]	0.756
Height, cm	52.0 [50.0–55.0]	53.0 [50.0–55.0]	52.0 [50.0–55.0]	0.586
CT dose-length product, mGy·cm	12.90 [8.59–24.00]	14.50 [9.00–27.57]	11.45 [8.42–23.24]	0.378

Data are median [IQR] or *n* (%). Weight was available in 165 patients (71/73 in the arch obstruction/hypoplasia group and 94/95 in the comparison group), height in 164 (71/73 and 93/95), and dose-length product in 165 (70/73 and 95/95). Continuous variables were compared using Mann–Whitney U tests and categorical variables using Fisher’s exact test. Arch/isthmic hypoplasia required explicit documentation in the original CT report; no uniform study-wide z-score or aortic-ratio threshold was applied.

**Table 2:** Quantitative PDA geometry according to CT-documented aortic arch status.

Parameter	CT-Documented Arch Obstruction or Hypoplasia	No CT-Documented Arch Obstruction/Hypoplasia	Raw *p*	Holm *p* *	Rank-Biserial *r*
Ductal Length-to-Diameter Index (LDI) †	2.04 [1.39–2.84]	3.51 [2.23–4.89]	<0.001	—	−0.473
PDA length, mm †	10.00 [8.45–14.00]	15.00 [9.75–19.50]	<0.001	0.003	−0.313
Aortic-end diameter, mm	5.20 [4.00–6.75]	4.80 [3.45–6.10]	0.106	0.424	0.146
Pulmonary-end diameter, mm	6.10 [4.00–7.50]	4.00 [2.90–5.70]	<0.001	<0.001	0.387
Aortic-to-pulmonary ratio	0.95 [0.75–1.07]	1.10 [0.90–1.40]	<0.001	0.001	−0.334
Mid-to-end caliber ratio †	0.89 [0.60–1.05]	0.95 [0.70–1.24]	0.131	0.424	−0.137
Caliber uniformity index †	0.62 [0.47–0.77]	0.62 [0.48–0.75]	0.925	0.925	−0.009
End-asymmetry index	0.21 [0.07–0.37]	0.21 [0.11–0.46]	0.423	0.846	−0.072

* Holm adjustment was applied across the seven secondary morphometric comparisons; LDI was not included in that multiplicity family. † PDA length and D_Mid were unavailable in one patient from the arch obstruction/hypoplasia group. Negative rank-biserial values indicate lower values in the CT-documented arch obstruction/hypoplasia group.

**Table 3:** Interobserver reproducibility of PDA measurements and derived geometric indices.

Parameter	ICC(A,1) (95% CI)	Lin CCC	Bias *	95% LoA	MAE
Aortic-end diameter, mm	0.911 (0.785–0.948)	0.908	−0.22	−1.84 to 1.39	0.65
Pulmonary-end diameter, mm	0.865 (0.676–0.961)	0.861	−0.21	−2.22 to 1.79	0.67
Mid-duct diameter, mm	0.905 (0.770–0.966)	0.902	0.01	−1.55 to 1.57	0.54
PDA length, mm	0.774 (0.522–0.936)	0.768	0.62	−7.66 to 8.90	2.50
Ductal Length-to-Diameter Index (LDI)	0.843 (0.685–0.954)	0.839	0.24	−1.98 to 2.45	0.64
Aortic-to-pulmonary ratio	0.823 (0.483–0.919)	0.818	−0.02	−0.51 to 0.46	0.18
Mid-to-end caliber ratio	0.697 (0.503–0.845)	0.690	−0.04	−0.55 to 0.48	0.18
Caliber uniformity index	0.769 (0.538–0.885)	0.764	0.06	−0.18 to 0.30	0.11
End-asymmetry index	0.680 (0.258–0.868)	0.673	−0.06	−0.38 to 0.27	0.13
Categorical morphology (deterministic profile)	κ = 0.460 (0.237–0.665)	—	Exact agreement	56.7% (40.0–73.3%)	—

* Bias = Reader 2 − Reader 1. ICC = two-way random-effects, absolute-agreement, single-measure intraclass correlation coefficient; CCC = Lin concordance correlation coefficient; LoA = limits of agreement; MAE = mean absolute error. Confidence intervals were calculated using 10,000 paired patient-level percentile bootstrap resamples with fixed seed 20260818. Categorical sensitivity analyses are reported in Table S3.

## Data Availability

The analysis code is provided as Supplementary Code S1 under an MIT License. De-identified data supporting the findings are available from the corresponding authors upon reasonable request, subject to institutional and ethical restrictions. A fully synthetic demonstration dataset is included with Supplementary Code S1 to permit end-to-end execution of the analysis pipeline without access to patient data.

## Supplementary Materials

The following supporting information can be downloaded at: https://www.mdpi.com/article/10.3390/jcm15208017/s1, Table S1: CT-based diagnostic adjudication of the 168 patients of the final cohort; Table S2: de-identified interobserver measurements and categorical profiles of both readers in the 30-patient interobserver substudy; Table S3: agreement of categorical morphology profiles between the two readers; Table S4: sensitivity, robustness, and exploratory model analyses; Table S5: rules of the rule-based categorical morphology profiles; Figure S1: LDI across congenital heart disease physiological groups; Figure S2: Bland–Altman agreement for LDI in the interobserver substudy; Supplementary Code S1: executable Python analysis pipeline and synthetic demonstration data.

## Abbreviations

The following abbreviations are used in this manuscript:

AIC	Akaike information criterion
AUC	area under the receiver operating characteristic curve
CCC	concordance correlation coefficient
CHD	congenital heart disease
CT	computed tomography
DLP	dose-length product
ECG	electrocardiogram
ICC	intraclass correlation coefficient
IQR	interquartile range
LDI	ductal length-to-diameter index
LoA	limits of agreement
MAE	mean absolute error
OR	odds ratio
PDA	patent ductus arteriosus
PGE1	prostaglandin E1
