# Supplementary material for: Quantitative CT Geometry of the Patent Ductus Arteriosus in Infants with Congenital Heart Disease: Association with Aortic Arch Obstruction or Hypoplasia
Source: J Clin Med. 2026 Oct 16;15(20):8017. doi: 10.3390/jcm15208017 (PMC13706272; doi:10.3390/jcm15208017)
Supplement: Supplementary file 1 [file jcm-15-08017-s001.zip › Supplementary_Materials.pdf]

Supplementary Materials

Quantitative CT Geometry of the Patent Ductus Arteriosus in Infants with Congenital Heart Disease: Association with Aortic Arch Obstruction or Hypoplasia

Marian Pop, Liel Bargutin, Răzvan-Andrei Licu, Marian Rotariu, Diana-Ramona Iurian, Tania Negovan and Horațiu Suciu

Contents

- **Table S1**: CT-based diagnostic adjudication (cited in Section 2.5).
- **Table S2**: de-identified interobserver measurements and categorical profiles (cited in Sections 2.6 and 3.6).
- **Table S3**: agreement of categorical morphology profiles (cited in Sections 2.6 and 3.6 and in the footnote of Table 3).
- **Table S4**: sensitivity, robustness, and exploratory model analyses (cited in Sections 2.7 and 3.5).
- **Table S5**: rules of the rule-based categorical morphology profile (cited in Section 2.6).
- **Figure S1**: LDI across congenital heart disease physiology groups (cited in Section 3.4).
- **Figure S2**: Bland–Altman agreement for LDI in the interobserver substudy (cited in Section 3.6).
- **Supplementary Code S1**: executable Python analysis pipeline and synthetic demonstration data (cited in Section 2.7 and in the Data Availability Statement).

Tables S1–S5 are provided as a single Excel workbook (one sheet per table, preceded by a Contents sheet with notes and abbreviations); Supplementary Code S1 is provided as a ZIP archive. Both files are supplied separately from this document.

Supplementary Tables S1–S5 (Excel workbook)

- **Table S1.** CT-based diagnostic adjudication of the 168 patients of the final cohort: case definition (CT-documented arch obstruction/hypoplasia), diagnostic subtype, four-level diagnostic group, three-level physiology group, and CT diagnosis (original Romanian report wording, with English translation). Underlying data for Section 3.1 and Figure 2 of the main text.
- **Table S2.** De-identified measurements (PDA length, aortic-end, mid, and pulmonary-end diameters) and categorical profiles of both readers in the 30-patient interobserver substudy. Underlying data for Table 3 of the main text and Figure S2.
- **Table S3.** Agreement of algorithmic and reader-assigned categorical morphology profiles between the two readers, including categorical sensitivity analyses (10,000 paired patient-level bootstrap resamples; seed 20260818).
- **Table S4.** Sensitivity, robustness, and exploratory model analyses: adjusted logistic regression, restricted and broader comparator analyses, three-group and pairwise comparisons, component model, age-stratified and interaction analyses, model comparison, spline test, alternative adjudication of borderline CT wording, conservative case definitions, and exploratory discrimination. Full outputs of the analyses summarized in Sections 3.2, 3.4, and 3.5 of the main text.
- **Table S5.** Rules of the rule-based categorical morphology profile (complex/atypical, window-short, hourglass, tubular, conical-aortic, conical-pulmonary, mixed), evaluated in order with the first matching rule applied, together with definitions, boundary handling, and verification: applied to the measurements in Table S2, the rules reproduce all 60 stored algorithmic profiles.

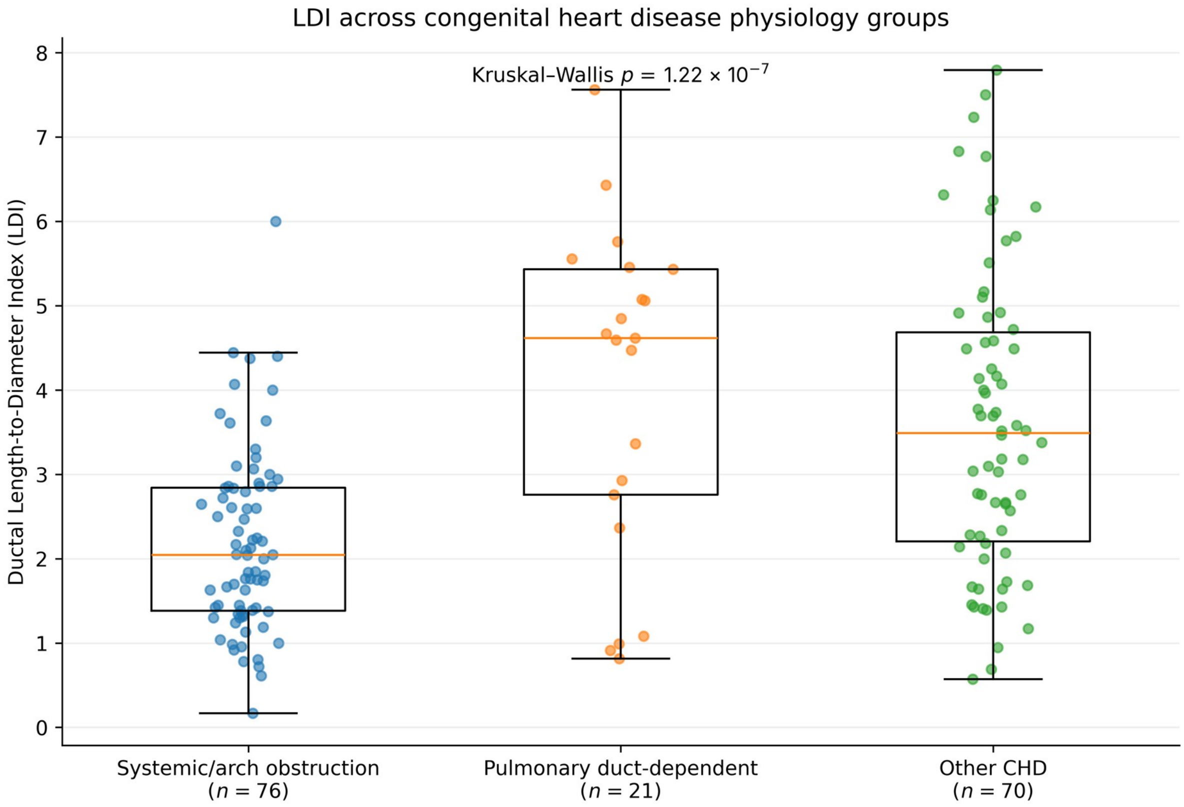

Figure S1. Ductal Length-to-Diameter Index (LDI) across congenital heart disease physiology groups: systemic/arch obstruction (LDI available in 76 patients), pulmonary duct-dependent disease (21 patients), and other congenital heart disease (70 patients). Individual observations are superimposed on boxplots; boxes represent the interquartile range (IQR), the orange horizontal line indicates the median, and whiskers extend to the most extreme values within 1.5 × IQR of the box. Kruskal–Wallis $p = 1.22 \times 10^{-7}$. After Holm correction, systemic/arch obstruction differed from pulmonary duct-dependent disease (adjusted $p = 0.00027$) and from other congenital heart disease (adjusted $p = 1.03 \times 10^{-6}$), whereas pulmonary duct-dependent disease and other congenital heart disease did not differ significantly (adjusted $p = 0.258$). Related to Section 3.4 of the main text. CHD, congenital heart disease; LDI, Ductal Length-to-Diameter Index.

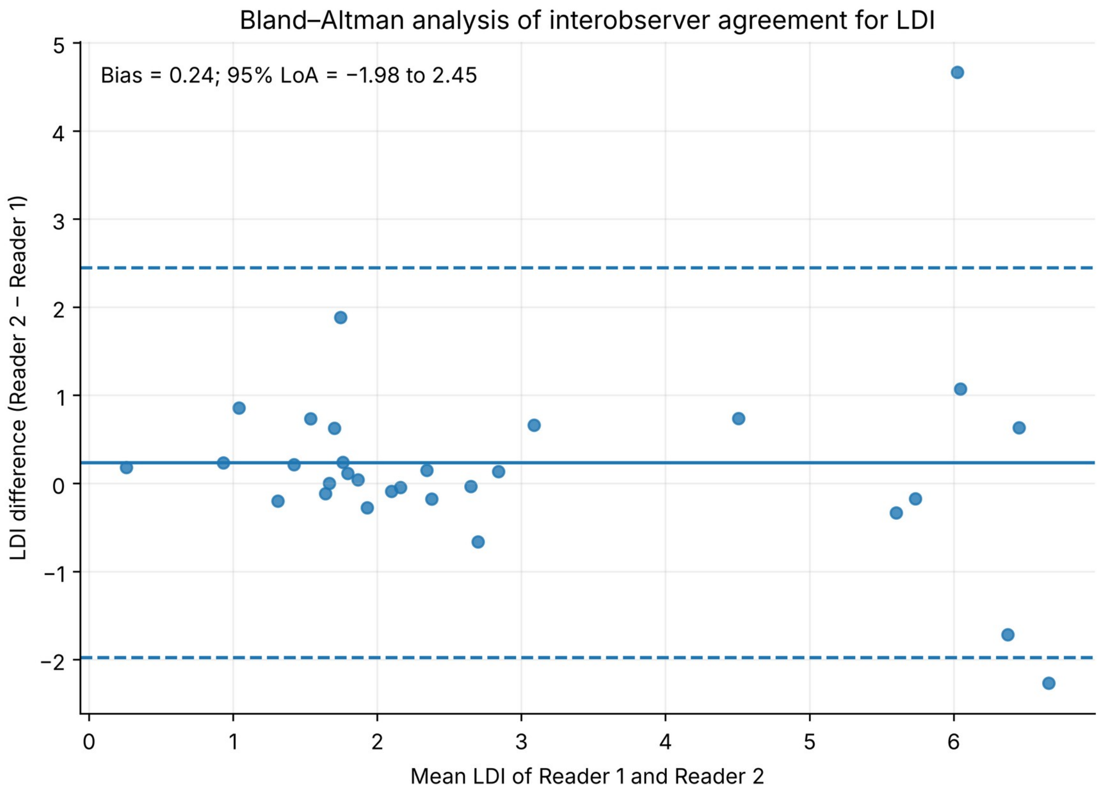

Figure S2. Bland–Altman analysis of interobserver agreement for the Ductal Length-to-Diameter Index (LDI) in the 30-patient interobserver substudy (Section 3.6 of the main text; individual measurements in Table S2). Differences (Reader 2 − Reader 1) are plotted against the mean of the two readers. The solid line indicates the mean bias (0.24) and the dashed lines the 95% limits of agreement (−1.98 to 2.45). LDI, Ductal Length-to-Diameter Index; LoA, limits of agreement.

Supplementary Code S1

ZIP archive containing the Python analysis pipeline (scripts 01–07, run_all.py, and verify_reproduction.py; script 07 checks the profile rules of Table S5 against Table S2), a README with instructions, the list of required packages with minimum versions (requirements.txt), the MIT License, and a fully synthetic demonstration dataset that allows the pipeline to be executed without access to patient data. The scripts read Tables S1 and S2 directly from the supplementary workbook. Full numerical reproduction requires the de-identified study data described in the Data Availability Statement.
